# Sexual abuse and substance use before or during sex among transgender women in Brazil

**DOI:** 10.1590/0102-311XEN105425

**Published:** 2026-03-16

**Authors:** Rafael Steffens Martins, Jonatan da Rosa Pereira da Silva, Daniela Riva Knauth, Andrea Fachel Leal, Luciana Barcellos Teixeira, Ines Dourado, Laio Magno, Ana Rita Coimbra Motta-Castro, Rita Bacuri de Queiroz, Maria Amélia de Sousa Mascena Veras

**Affiliations:** 1 Universidade Federal do Rio Grande do Sul, Porto Alegre, Brasil.; 2 Universidade Federal da Bahia, Salvador, Brasil.; 3 Universidade do Estado da Bahia, Salvador, Brasil.; 4 Universidade Federal de Mato Grosso do Sul, Campo Grande, Brasil.; 5 Fundação Oswaldo Cruz, Manaus, Brasil.; 6 Santa Casa de São Paulo, São Paulo, Brasil.

**Keywords:** Transgender, Sexual Abuse, Illicit Drugs, Transgênero, Abuso Sexual, Drogas Ilícitas, Transgénero, Abuso Sexual, Drogas Ilícitas

## Abstract

Transgender women and *travestis* (TWT) experience high rates of sexual violence and substance use, but few studies explore drug use specifically before or during sex. As such, this study investigated the association between lifetime sexual abuse and drug use before or during sex among TWT in Brazil. TransOdara was a cross-sectional study conducted in five Brazilian cities using respondent-driven sampling from 2019 to 2021. A total of 1,317 TWT completed a questionnaire aided by trained interviewers. Poisson regression was used to estimate associations between abuse history and substance use (marijuana, cocaine or crack) before or during sex, adjusting for confounders. Over half (53.9%) of the participants reported sexual abuse or attempted abuse, and 36.7% reported substance use before or during sex. Abuse history was associated with a higher prevalence of substance use (adjusted PR = 1.17; 95%CI: 1.01-1.35). History of sex work was also associated (PR = 2.02), whereas high school completion proved a protective factor (PR = 0.82). Sexual abuse is significantly associated with substance use in sexual contexts among TWT. Public health strategies must address trauma, substance use, and social vulnerabilities in this population, bringing together the efforts of public health and human rights.

## Abstract (pt)

Mulheres transgênero e travestis (MTTs) enfrentam altas taxas de violência sexual e uso de substâncias, mas poucos estudos exploram o uso de drogas especificamente antes ou durante o sexo. O objetivo foi investigar a associação entre o abuso sexual ao longo da vida e o uso de drogas antes ou durante o sexo entre as MTTs no Brasil. O TransOdara foi um estudo transversal realizado em cinco cidades brasileiras utilizando *respondent-driven sampling* entre 2019 e 2021. Um total de 1.317 MTTs preencheram um questionário aplicado por entrevistadores treinados. A regressão de Poisson foi usada para estimar associações entre o histórico de abuso e uso de substâncias (maconha, cocaína ou *crack*) antes ou durante o sexo, com ajustes para fatores de confusão. Mais da metade (53,9%) dos participantes relatou abuso sexual ou tentativa de abuso, e 36,7% relataram uso de substâncias antes/durante o sexo. O histórico de abuso esteve associado a uma prevalência maior de uso de substâncias (RP ajustada = 1,17; IC95%: 1,01-1,35). O histórico do trabalho sexual comercial também estava associado (RP = 2,02), enquanto a conclusão do Ensino Médio foi fator de proteção (RP = 0,82). O abuso sexual está significativamente associado ao uso de substâncias em contextos sexuais entre MTTs. As estratégias de saúde pública devem abordar traumas, uso de substâncias e vulnerabilidades sociais nessa população, unindo os esforços da saúde pública e dos direitos humanos.

## Abstract (es)

Las mujeres transgénero y travestis (MTT) se enfrentan a altas tasas de violencia sexual y consumo de sustancias, pero pocos estudios exploran el consumo de drogas específicamente antes o durante las relaciones sexuales. El objetivo del estudio era investigar la asociación entre el abuso sexual a lo largo de la vida y el consumo de drogas antes o durante las relaciones sexuales entre las MTT en Brasil. TransOdara fue un estudio transversal realizado en cinco ciudades brasileñas utilizando *respondent-driven sampling* entre 2019 y 2021. Un total de 1.317 MTT completaron un cuestionario con entrevistadores capacitados. Se utilizó la regresión de Poisson para estimar las asociaciones entre los antecedentes de abuso y el consumo de sustancias (marihuana, cocaína o *crack*) antes o durante las relaciones sexuales, ajustando los factores de confusión. Más de la mitad (53,9%) de las participantes informaron haber sufrido abusos sexuales o intentos de abuso, y el 36,7% informaron haber consumido sustancias antes o durante las relaciones sexuales. Los antecedentes de abuso se asociaron con una mayor prevalencia del consumo de sustancias (RP ajustado = 1,17; IC95%: 1,01-1,35). Los antecedentes de trabajo sexual comercial también se asociaron (RP = 2,02), mientras que haber completado la enseñanza secundaria fue un factor protector (RP = 0,82). El abuso sexual se asocia significativamente con el consumo de sustancias en contextos sexuales entre las MTT. Las estrategias de salud pública deben abordar el trauma, el consumo de sustancias y las vulnerabilidades sociales de esta población, unificando los esfuerzos de la salud pública y los derechos humanos.

## Introduction

History of sexual abuse is associated with many negative psychosocial outcomes such as depression, anxiety, and stress ^1^. Additionally, several studies have shown a link between a history of sexual abuse and increased consumption or dependence on drugs ^1^ ^,^ ^2^ ^,^ ^3^.

A study conducted in the United States revealed that sexual diverse females and males are more likely to report sexual abuse compared with their heterosexual counterparts ^4^. The vulnerability of gender minorities, including transgender women and *travestis* (TWT), to sexual abuse and its detrimental health effects can be further increased by some factors that are more common within these populations, including a history of sex work ^5^.

Data from a study with 1,317 TWT including all Brazilian regions indicate a lifetime prevalence of 52% for marijuana, 42.6% for cocaine and 13.9% for crack ^6^. In contrast, the estimated prevalence of these substances in the Brazilian general population is 7.7% for marijuana, 3.1% for cocaine and 0.9% for crack ^7^. Other studies show disproportionately high rates of psychoactive substances use among transgender individuals compared with cisgender ones ^8^. This significant disparity in substance use may be associated with the different forms of discrimination and trauma experienced by this population throughout their lives, though this remains underexplored in scientific studies.

While the association between sexual abuse and drug use is well-documented in the literature ^3^, few studies examine specifically substance use before or during sexual activity. Research on this topic is even more limited when it comes to transgender women ^3^ ^,^ ^9^ in the Brazilian context. The practice of chemsex has been associated with increased vulnerability to sexually transmitted infections (STIs), decreased condom use, and other negative health outcomes ^10^ ^,^ ^11^. Considering this gap, this study investigated the association between a history of sexual abuse or attempted sexual abuse and drug use before or during sex among transgender women in Brazil.

## Methods

The study data originates from the TransOdara study conducted from 2019 to 2021 across capital cities representing Brazil’s five distinct regions: São Paulo (São Paulo State, Southeast), Campo Grande (Mato Grosso do Sul State - Central-West), Manaus (Amazonas State - North), Porto Alegre (Rio Grande do Sul State - South), and Salvador (Bahia State - Northeast). This cross-sectional study was primarily engaged in estimating the prevalence of syphilis and other STIs among TWT in Brazil ^12^.

A sample size of 1,280 TWT was calculated a priori to estimate the prevalence of syphilis with standard errors for each site. Participants were recruited using the respondent-driven sampling (RDS) method in which “seeds” were selected in a formative phase of the study to initiate the study network ^13^. Each seed could invite five known TWT, who would also receive five invitations each, creating a recruitment chain. Those invited were then asked to complete a questionnaire, aided by trained researchers, covering various aspects including sociodemographic information, experiences of violence, substance use, and sexual behaviors.

RDS was employed in the TransOdara study to sample a population that is difficult to access using other sampling methods. However, the primary aim of these analyses is not to produce representative and generalizable prevalence estimates. Instead, the focus is on examining the association between drug use and sexual abuse among transgender women. As a result, the data remain unweighted to avoid introducing significant variance from RDS adjustments, since variable-specific weights may not be appropriate for multivariate analyses ^14^. For further details, see the methodological article ^12^.

A standardized, interviewer-administered survey was used to gather sociodemographic data and included over 200 items covering gender-affirming procedures, experiences of stigma and discrimination, incarceration history, mental health, alcohol and other substance use, sexual behaviors, access to healthcare and HIV/STI testing, pre- and post-exposure prophylaxis for HIV prevention, and STI symptoms in the past six months and at the study visit. Face-to-face interviews were conducted by well-trained researchers using laptops. All data, including responses from interviewer-administered questionnaires and case report forms, were collected and managed as a single dataset using REDCap (https://redcapbrasil.com.br/) ^15^. Participants were reimbursed for food and transportation expenses and were required to provide informed consent to participate.

Descriptive analyses characterized the sample by exploring sociodemographic variables such as age (18-24, 25-35, 36-45, 46 years or more), race/color (White, Black, Mixed-race, Yellow/Indigenous), schooling level (incomplete middle school, complete middle school/incomplete high school, complete high school, higher education), and marital status (without a regular partner, dating, married or living together), as well as characteristics related to abuse history, including sexual abuse or attempted abuse (yes, no) and forced first intercourse (yes, no), history of commercial sex (yes, no) and drug use before or during sex in the last year (yes, no). The variables were presented as absolute and relative frequencies. Regarding the sociodemographic variable race/color, skin color was self-reported using the official categories of the Brazilian Institute of Geography and Statistics (IBGE, acronym in Portuguese): *branco* (White), *preto* (Black), *pardo* (Mixed-race), *amarelo* (Yellow), and *indígena* (Indigenous). The category *pardo* includes individuals who identify as having mixed ancestry, including varying degrees of Indigenous, African, and European heritage. In Brazilian Portuguese, the term *negro* is used to collectively refer to individuals who identify as *preto* or *pardo*, and these categories are presented as such here.

For this analysis, the main exposure variable “history of sexual abuse or attempted abuse” was constructed from the question: “Has anyone ever tried or forced you to have sex against your will at any point in your life?”. Additionally, participants who reported having had a nonconsensual first sexual experience were also considered as having a history of sexual abuse in their lifetime. The outcome variable “drug use before or during sex in the last year” was defined based on participants’ self-reported use of psychoactive substances in sexual contexts. Participants were asked: “In the past 12 months, have you used any substances before or during sex? If yes, which substances did you use?”. Drug use was considered positive when participants reported using marijuana, cocaine, or crack in these situations. This variable reflects substance use specifically associated with sexual activity, rather than general or recreational consumption. The variable was treated as binary (yes, no), indicating whether the participant had engaged in drug use before or during sex at least once in the past year.

Association between history of sexual abuse and drug use before or during sex (outcome) was estimated by Poisson regression with robust variance. Following a univariable model, a secondary analysis adjusted for confounders such as race/color (Black, Mixed-race/others), schooling level (secondary education completed/uncompleted), marital status (without a regular partner/dating or married), and history of commercial sex (yes or no) was conducted. All analyses were conducted with a 5% significance level on the SPSS, version 26 (https://www.ibm.com/).

The study protocol was approved by the Research Ethics Committee of Santa Casa de Misericórdia de São Paulo (CAAE 05585518.7.0000.5479), as well as by the ethics committees of all participating institutions. Further details on the TransOdara study methodology can be found in the methodological manuscript ^12^.

## Results

The final study sample consisted of 1,317 TWT. Nearly 42.6% of the participants were aged 25 to 35, whereas 23.9% had 18 to 24 years old. Of the sample, 44.1% self-identified as Mixed-race and 26.7% as Black. Regarding schooling level, descriptive analysis revealed that only 16.9% entered tertiary education, whereas 41.9%, completed secondary education. As for marital status, most participants consider themselves as single (71.7%). Approximately 73.7% of the participants have engaged in sex in exchange for money or goods at some point in their lives, and 36.7% used substances before or during sex in the past year. As for variables related to sexual abuse, 53.9% have a history of sexual abuse or attempted abuse, and 16% experienced a nonconsensual first intercourse (Table 1).

**Table 1:** Behavior, sociodemographic characteristics, and sexual abuse history among transgender women.

Characteristics	% (n)
Age group (years)	
18-24	23.9 (315)
25-35	42.6 (561)
36-45	21.3 (280)
≥ 46	12.2 (161)
Race/Color	
White	25.7 (336)
Black	26.7 (349)
Mixed-race	44.1 (575)
Other (Yellow/Indigenous)	3.4 (45)
Schooling level	
Incomplete middle school	17.8 (234)
Complete middle school/Incomplete high school	23.4 (307)
Complete high school	41.9 (550)
Higher education	16.9 (222)
Marital status	
Without a regular partner	71.7 (942)
Dating	13.9 (182)
Married or living together	14.4 (189)
History of commercial sex	
Yes	73.7 (970)
No	26.3 (347)
Drug use before or during sex in the last year	
Yes	36.7 (483)
No	63.3 (834)
History of sexual abuse or attempted abuse	
Yes	53.9 (706)
No	46.1 (603)
First sexual intercourse	
Consensual	84.0 (1,095)
Forced	16.0 (208)

Estimation from the univariate model using regression analysis revealed that a history of sexual abuse was positively associated with a higher prevalence of substance use before or during sex in the past year (prevalence ratio - PR = 1.27; 95% confidence interval - 95%CI: 1.10-1.47). In the multivariate analysis, a history of substance use before or during sex remained associated with a history of sexual abuse, being higher among those who experienced sexual abuse during their lifetime (PR = 1.17; 95%CI: 1.01-1.35). Among the adjusted variables, a history of sex work increased the prevalence of substance use before or during sex (PR = 2.02; 95%CI: 1.60-2.55), whereas completing high school acted as a protective factor (PR = 0.82; 95%CI: 0.71-0.95) (Table 2).

**Table 2:** Comparison of behavior and sociodemographic characteristics among transgender women, according to history of sexual abuse.

Characteristics	PR (95%CI)	
	Model 1	Model 2
History of sexual abuse or attempted abuse		
No	1.00	1.00
Yes	1.27 (1.10-1.47)	1.17 (1.01-1.35)
History of commercial sex		
No	-	1.00
Yes	-	2.02 (1.60-2.55)
Complete high school completed or more		
No	-	1.00
Yes	-	0.82 (0.71-0.95)
Marital status		
Without a regular partner	-	1.00
Dating or married	-	1.10 (0.95-1.28)

95%CI: 95% confidence interval; PR: prevalence ratio.

## Discussion

Unlike other studies that have investigated associations with substance use in general, our research specifically explored drug use before or during sex. Results revealed an association between a history of sexual abuse and attempted sexual abuse with drug use before or during sex. In our sample, TWT with a history of sexual violence were 17% more likely to use substances before or during sex.

This topic is particularly relevant given the high prevalence of sexual abuse history among this population (approximately 53.9% of the sample). Prior research from TransOdara has highlighted the associated vulnerability and limited institutional support faced by Brazilian TWT, indicating that despite repeated occurrences of sexual violence, most did not seek health services, report the incident, or turn to family or friends ^16^. Other international and national studies have reported similar prevalence of sexual abuse among this population ^17^ ^,^ ^18^ and found that compared with cisgender people, trans people have a higher history of experiencing many types of violence, including sexual ^5^. Additionally, this population also uses more illicit substances compared with cisgender people ^5^ ^,^ ^8^ ^,^ ^19^.

Understanding the relation between sexual violence and substance use is essential since these experiences are more common among this population. Several studies describe similar findings, but they generally investigate substance use without considering whether its usage occurred before sexual activity, highlighting the association between a history of sexual violence and drug use ^17^ ^,^ ^18^ ^,^ ^19^ ^,^ ^20^ ^,^ ^21^. In investigating a sample of cisgender women who consumed alcohol and other drugs, a study found that those with a history of childhood sexual abuse exhibited higher levels of anxiety, self-harm, eating disorders, and sexual dysfunction ^22^. According to Magalhães et al. ^18^, a history of violence may be associated with a higher prevalence of overall drug use as a mechanism for coping with trauma among transgender women. Said correlation proves greater when substance use is specifically related to sexual activity, as substance use can serve as an outlet, inducing relaxation, disinhibition, and forgetfulness ^23^.

Sexual abuse leaves psychological wounds that negatively impact the mental health of transgender individuals, increasing the likelihood of depression, anxiety, and suicide attempts. Moreover, victims often face social stigmas and discrimination which can increase isolation and hinder access to support networks ^24^ ^,^ ^25^ ^,^ ^26^. Experiences of violence not only compromise TWT’s mental health but also undermine their ability to establish healthy relationships, which can in turn increase drug use.

Our findings also reveal that a history of commercial sex is associated with substance use before or during sex. Some transgender women involved in commercial sex use drugs to cope with work-related trauma, whereas others enter sex work to finance their substance use in vulnerable situations ^5^ ^,^ ^20^. Qualitative studies with TWT found that it is common for clients to offer extra payment to use substances with sex workers ^5^. Additionally, transgender women with a history of commercial sex are at greater risk of sexual abuse ^5^ ^,^ ^27^.

Having higher education proved to be a protective factor against substance use before sex in our study. This lower prevalence of substance use among TWT with higher education may be explained not only by increased knowledge, but also by enhanced social capital and access to supportive networks. Education can help TWT handle social and structural challenges more effectively, making them less likely to use substances to cope with trauma or stress from the many violences experienced during life, including sexual violence. In this regard, schooling serves not only as a source of information, but also as a buffer against vulnerability, particularly in a population that faces multiple barriers to educational access, including family rejection. This protective role proves particularly relevant given the educational disparities in our sample, in which over 40% of participants had not completed high school.

Although marijuana, cocaine, and crack are all illegal in Brazil (including differing legal penalties under *Law n. 11,343/2006* ^28^) and vary in their effects and social contexts, they were grouped into a single variable representing drug use before or during sex as our purpose was not to analyze the specific psychoactive effects of each substance, but rather the practice of using drugs in sexual contexts. Similar approaches have been adopted in other studies examining the relation between psychoactive substance use and sexual behavior, and previous research has shown that each of these drugs can individually be associated with higher sexual risk behaviors ^29^ ^,^ ^30^ ^,^ ^31^.

Our results on drug use before or during sex should be interpreted with caution, as we did not assess the frequency or motivations for such use. What the variable captures is psychoactive drug use in sexual contexts without distinguishing between occasional and regular use, which may influence its association with sexual risk behaviors. Additionally, its cross-sectional design does not allow determining the temporal relation between sexual abuse history and substance use, limiting causal inferences. On the other hand, one strength is our focus on understanding the correlation between sexual abuse history and substance use specifically when said use occurred in sexual contexts, as many studies evaluate substance use in general. Moreover, this study had a large sample of transgender women, a hard-to-reach population, from all five regions of Brazil.

Importantly, data collection started in 2019 and was extended until 2021 due to the COVID-19 pandemic. Despite significant challenges posed by the pandemic and the fieldwork targeting a vulnerable population, the TransOdara study was successfully conducted. Its findings should be interpreted considering the potential impact of the pandemic and social isolation on sexual behavior and substance use ^12^.

In conclusion, our results highlight the significant association between sexual abuse history and attempted sexual abuse with substance use before or during sex among transgender women in Brazil. This correlation underscores the need for targeted interventions addressing both trauma and substance use in this vulnerable population, bringing together the efforts of public health and human rights.

## Data Availability

The research data are available upon request to the corresponding author.
